# A Correlational Study on Professional Identity and Self-Efficacy Among Nursing Students

**DOI:** 10.7759/cureus.67508

**Published:** 2024-08-22

**Authors:** Aheli Mukherjee, Aarthi Saravanan, Abisha Kamaraj, Vijayalakshmi Radhakrishnan

**Affiliations:** 1 Nursing, SRM College of Nursing, SRM Institute of Science and Technology, Kattankulathur, IND

**Keywords:** self-confidence, correlational study, nursing students, self-efficacy, professional identity

## Abstract

Aim

To conduct a correlational study on professional identity and self-efficacy among nursing students

Background

Professional identity, in simple terms, refers to how one perceives oneself in relation to one’s profession. Self-efficacy is defined as people’s self-confidence in facing challenges and breaking through difficulties. A well-developed level of self-efficacy may enhance professional identity. This study sought to assess the professional identity and self-efficacy of student nurses enrolled at SRM College of Nursing, SRM Institute of Science and Technology, Kattankulathur, India, examine the relationship between professional identity and self-efficacy, and explore how self-efficacy and professional identity levels relate to demographic variables.

Methodology

A descriptive research design was utilized to assess the professional identity as well as the self-efficacy of 202 student nurses. The subjects were surveyed using the General Self-Efficacy Scale and Professional Identity Scale for Nursing Students questionnaires to analyze their levels of self-efficacy and professional identity, respectively.

Result

The results indicate that among the 202 students surveyed, 102 (50.5%) possess a moderate level of self-efficacy, and 71 (35.1%) possess a moderate level of professional identity. A strong positive correlation was found between professional identity and self-efficacy (r=0.489), implying that the student nurses with a prominent degree of self-efficacy have a compelling degree of professional identity and vice-versa.

Conclusion

In this investigation, most of the students demonstrated moderate levels of self-efficacy as well as professional identity. Additionally, a robust correlation was observed between self-efficacy and professional identity.

## Introduction

A global shortfall of 5.9 million qualified nurses has been predicted by prominent healthcare bodies like the World Health Organization (WHO) and the International Council of Nurses (ICN). The greatest shortfalls are in the Eastern Mediterranean locale, the African continent, and the Southeast Asian region. Some regions of Latin America also have nursing shortages. To combat the evidential shortage, there needs to be an increase of about 8% per year in the aggregate quantity of nursing graduates as well as an improvement in their retention rates and employment abilities [1,2]. However, simply increasing the number of nurses without considering their competency would be misguided. Therefore, it is crucial to focus on two aspects of quality assurance: Professional Identity and Self-Efficacy.

People's self-confidence in tackling risks and overcoming significant obstacles has been referred to as self-efficacy. A well-developed self-efficacy level improves job satisfaction and intention to stay in a profession. It helps to tackle challenges rather than put them off, which is an essential skill in every profession, especially in nursing [3,4]. Furthermore, self-efficacy may significantly improve professional identity [5]. Professional identity, in simple terms, refers to how one perceives self in relation to one’s profession. It has been shown to have a confirmed correlation with job satisfaction, thus facilitating job retention [4].

Self-efficacy has been shown to be excellent at enabling nursing students to tackle the challenges in clinical fields and improve their credibility and competence [6,7]. When self-efficacy is developed properly, the intention to stay in a profession and job satisfaction levels are also affected positively [7]. Reports further show that low self-efficacy levels lead to avoidance of tasks that invoke a sense of failure. 

Nurses, with the convergence of Western and Indian perspectives, have undertaken the arduous task of creating a new identity for themselves in Indian society and especially within the Medical community. With migration being a huge part of the Indian nursing system, there needs to be increased scrutiny of the human resource policy for health since migration has invariably contributed to the shortfall of nursing personnel in India. Therefore, improving working conditions is necessary to stave off the shortage. Concurrently, enhancing professional identity and self-efficacy levels is essential to build a workforce capable of managing the stress of working in a densely populated country like India [8]. 

Studies have also shown that any downturn in the salience of professional identity can discount a profession's value and the advice and ideas given by said professionals. Furthermore, in India, the nursing profession already has a decreased social value compared to medicine, which can further diminish the perceived professional identity of the personnel involved. Decreased levels of self-efficacy, professional identity or both are identified as causes of burnout and dissatisfaction with employment, according to many researches [4]. This in turn perpetuates a cycle of increased stress in nurses and an increased nurse turnover [9].

Research indicates that nursing students were able to manage the recent coronavirus pandemic and developed resilience through active engagement, spiritual pursuits, and levity. During the pandemic, it was strongly recommended that individuals prioritize stress management in order to promote mental health [10]. It was found that nurses' self-efficacy and anxiety during the pandemic improved with enough targeted COVID-19 training. Although many nurses have achieved improvement in self-efficacy in recent years, personal and professional concerns still cause anxiety. Thus it was recommended that hospitals should engage in comprehensive training programs to boost nurses' confidence and mental health, boosting their pandemic management [11]. Additionally, mentorship programs could be undertaken as research has shown that they help students overcome obstacles and build their professional identity and self-efficacy [10].

Moreover, reducing the turnover rate in the nursing profession requires effort to enhance work satisfaction and resilience. The ideal approach is to improve self-efficacy and professional identity. This is because self-efficacy can help upswing resilience by enabling them to tackle challenges head-on rather than avoiding them [4]. 

However, it is important to note that it takes time to improve self-efficacy, much more so in case of professional identity. Therefore, it is essential to start at the undergraduate level and work upwards. To do so, we first need to analyze current levels of self-efficacy and professional identity. Unfortunately, very little analysis has been done on self-efficacy and professional identity in India, more so in Tamil Nadu. So, this study aims to fill that lacuna as well as analyze any possible co-relationship between the two.

## Materials and methods

Research design

A quantitative research approach with a descriptive research design was adopted to assess the professional identity as well as the self-efficacy of student nurses at SRM College of Nursing. The SRM College of Nursing Ethical Committee approved the study with the approval number SRMCONIPAC-ST2023-001.

Sample size

We employed a convenience sampling strategy to select the sample. A total of 202 students from Bachelor of Science (BSc) Nursing Third and Fourth year and Diploma in General Nursing and Midwifery (DGNM) Second year students at SRM College of Nursing constituted the sample, in accordance with the following inclusion and exclusion criteria. Students studying in BSc Nursing Third and Fourth year and DGNM Second year students in SRM College of Nursing were included in the study and individuals who were absent during the study were excluded.

Instrument description

There are three parts to the questionnaire schedule:

Section A

This segment comprises Demographic Variables: Age, Gender, Course, Year, Family Income, Place of residence, Educational Backgrounds of the parents, and Occupation of the father and mother.

Section B

Self-efficacy: Schwarzer and Jerusalem created an instrument for self-evaluation, the General Self-efficacy Scale (1979) [12]. It has 10 items and is scored from 10 to 40 on a 4-point Likert scale. Its reliability has been confirmed with 0.76 to 0.90 Cronbach's alpha.

Section C

Professional identity: A measurement instrument for assessing the developing professional identity degree in student nurses, the Professional Identity Scale for Nursing Students (PISNS) was created by Hao et al. (2014) [13]. There are 17 elements across five factors, each appraised on a scale of five points. The split-half reliability was found to be 0.84 with Cronbach's alpha at 0.83.

Scoring and interpretation

Descriptive analysis was used to describe the demographic characteristics.

Self-efficacy: The sum of all components was used to compute the overall score. The total score for the General Self-Efficacy Scale (GSES) was quantified within a limit of 10 to 40, where a greater tally signifies a better degree of self-efficacy. On the GSES scale, 10 to 20 points are considered low, 21 to 30 points medium, and 31 to 40 points a high score.

Professional identity: Determining the sum of every item yields the overall score. The cumulative score for the Professional Identity Scale for Nursing Students (PISNS) varies between 17 and 85. An individual's favorable professional identity is proportional to their high score. The four levels make up the scale: The levels are as follows: low, which is less than the 34th percentile (< 61), moderate, which is between 61 and 71, and high, which is equal to or greater than the 67th percentile (≥71).

Data collection 

With the explicit consent of the Dean of the SRM College of Nursing, data collection was conducted. The research was conducted over one week, specifically from March 6, 2023, to March 10, 2023. Employing the convenience sampling method, 202 student nurses from SRM College of Nursing were selected in accordance with the predetermined inclusion and exclusion criteria. The investigator provided an explanation of the study's objectives. The students were assured of the strict confidentiality of the collected data. The subjects who were chosen for the study gave their written consent to participate in this research.

A self-introduction was provided to address the subjects selected. The intent of the study and the instruments used were explained briefly to the participants. The subjects were then surveyed for demographic information. To measure their self-efficacy, researchers utilized a structured questionnaire based on the GSES [12]. Similarly, to measure their professional identity, researchers utilized the PISNS [13]. The mean time required for an individual to submit the completed tools was about 15 minutes.

Statistical analysis

Frequency and percentage distributions were employed to evaluate the demographic variables. Standard deviation and mean were used to examine the degrees of self-efficacy and professional identity. Furthermore, the chi-square test was used to ascertain the relationship between demographic variables and study variables. Pearson's correlational analysis determined the relationship between the two study variables.

## Results

The evaluation of demographic variables pertaining to nursing students enrolled at SRM College of Nursing showed that out of the students surveyed, 131 (64.9%) were aged between 21-25 years, 136 (67.3%) students were females, 189 (93.6%) participants were in BSc Nursing course, 96 (47.5%) subjects were from BSc Fourth year and 149 (73.8%) students were day scholars. Furthermore, 140 (69.3%) pupils belonged to nuclear families, 115 (56.9%) subjects were the first child, 116 (57.4%) subjects were Hindu, 58 (28.7%) participants had a family income of Rs 25,000 to 50,000, 124 (61.4%) pupils lived in urban areas, 66 (32.7%) of their fathers had achieved a high school certificate, 66 (32.7%) of their mothers had achieved a high school certificate, 66 (32.7%) of their fathers were professional and 104 (51.5%) of their mothers were housewife/unemployed (Table 1).

**Table 1:** Demographic variables broken down by frequency and percentage BSc (N): BSc Nursing; DGNM: Diploma in General Nursing and Midwifery

S. No.	Demographic Variables	Class	No. of respondents	Percentage
1	Age (depicted in years)	15-20	69	34.2%
		21-25	131	64.9%
		25-30	1	0.5%
		30-35	1	0.5%
2	Gender	Male	66	32.7%
		Female	136	67.3%
3	Course	BSc Nursing	189	93.6%
		DGNM	13	6.4%
4	Year	BSc (N) 3rd year	93	46.0%
		BSc (N) 4th year	96	47.5%
		DGNM 2nd year	13	6.4%
5	Type of Student Accommodation	Hostel	53	26.2%
		Day Scholar	149	73.8%
6	Type of family	Nuclear family	140	69.3%
		Joint family	56	27.7%
		Extended family	1	0.5%
		Single parent family	5	2.5%
7	Order of child in the family	First child	115	56.9%
		Second child	52	25.7%
		Third child	29	14.4%
		Others	6	3.0%
8	Religion	Hindu	116	57.4%
		Christian	64	31.7%
		Muslim	20	9.9%
		Others	2	1.0%
9	Family Income	Less than 25,000	48	23.8%
		25,000 to 50,000	58	28.7%
		50,000 to 75,000	31	15.3%
		75,000 to 1,00,000	46	22.8%
		Above 1,00,000	19	9.4%
10	Place of residence	Urban	124	61.4%
		Semi-Urban	37	18.3%
		Rural	41	20.3%
11	Educational Background of the father	No formal education	14	6.9%
		Primary School Certificate	27	13.4%
		High School Certificate	66	32.7%
		Diploma	23	11.4%
		Graduate	46	22.8%
		Post Graduate	20	9.9%
		Others	6	3.0%
12	Educational Background of the mother	No formal education	16	7.9%
		Primary School Certificate	45	22.3%
		High School Certificate	66	32.7%
		Diploma	13	6.4%
		Graduate	35	17.3%
		Post Graduate	22	10.9%
		Others	5	2.5%
13	Occupation of father	Unemployed	6	3.0%
		Semi-skilled Worker	22	10.9%
		Skilled Worker	63	31.2%
		Professional	66	32.7%
		Business	40	19.8%
		Others	5	2.5%
14	Occupation of mother	Unemployed/ Housewife	104	51.5%
		Semi-skilled Worker	19	9.4%
		Skilled Worker	23	11.4%
		Professional	41	20.3%
		Business	12	5.9%
		Others	3	1.5%

An assessment of the self-efficacy of student nurses enrolled at SRM College of Nursing yielded the following results: 102 (50.5%) students demonstrated a medium degree of self-efficacy, 98 (48.5%) students showed a high level, and two (1.0%) students displayed a low level (Table 2).

**Table 2:** The degree to which student nurses possess self-efficacy

S. No.	Level of Self-efficacy	No. of students	Percentage
1.	Low	2	1.0%
2.	Medium	102	50.5%
3.	High	98	48.5%

After polling the professional identity for the available subjects, it was found that 71 (35.1%) students had a moderate level of professional identity, 67 (33.2%) students had a high level, and 64 (31.7%) students had a low level (Table 3).

**Table 3:** Proficiency in professional identity among student nurses

S. No.	Level of Professional Identity	No. of students	Percentage
1	Low	64	31.7%
2	Moderate	71	35.1%
3	High	67	33.2%

The underlying representation determines the inter-relationship of professional identity with self-efficacy in students at SRM College of Nursing (Table 4). Professional identity, with a mean score of 64.52 (SD=11.804), stood in stark contrast to the mean score for self-efficacy, which was 30.66 (SD=5.145). A highly substantial positive association (r=0.489) was found between self-efficacy and professional identity according to Karl Pearson's coefficient of correlation. This positive correlation implies that nursing students with prominent self-efficacy levels have compelling degrees of professional identity and vice-versa.

**Table 4:** Examining the relationship of self-efficacy with professional identity ** - Significant at 1% level; * - Significant at 5% level

S. No.	Variable	N	Mean	SD	r value	P value
1	Self-Efficacy	202	30.66	5.145	0.489	0.000**
2	Professional Identity	202	64.52	11.804		

**Figure 1:**
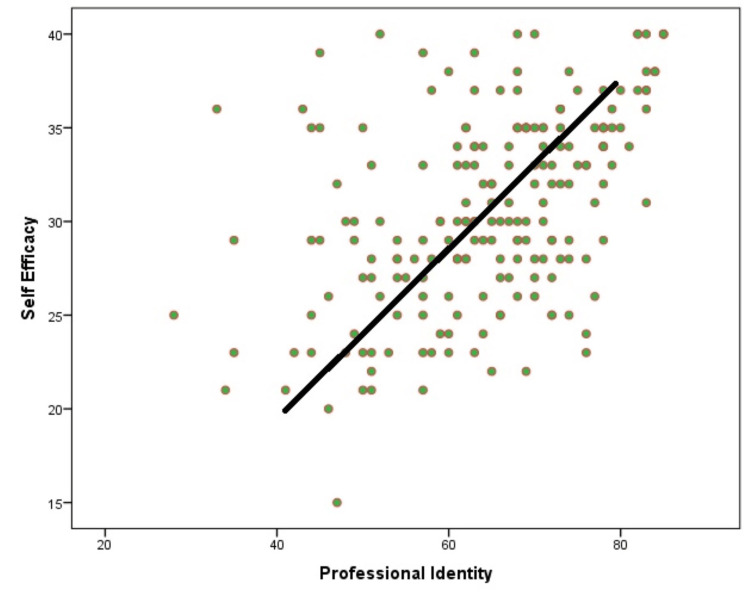
A scatter diagram depicting the hyperlink amid professional identity as well as self-efficacy of student nurses Figure Credit: Mr. Ravi Shankar

The scatterplot portraying professional identity and self-efficacy reveals a consistent upward trajectory from left to right, indicating a positive correlation between the two variables. Most of the data points exhibit a compact and consistent distribution around the "line of best fit", implying a robust and credible link between the variables. There is a minimal departure from this line, indicating that the values of one variable can be predicted with a high degree of accuracy based on the value of the other variable; that is, as self-efficacy grows, there is a corresponding increase in professional identity, indicating a positive connection amidst them (Figure 1). 

The relationship-engineering of selected demographic variables with professional identity, in addition to self-efficacy, was derived using the Chi-square test. The p-value for the parameter "Year of studying" is 0.000 (χ2=96.255), which is below the threshold of 0.01, indicating a high degree of statistical significance at the 1% level. Therefore, we can conclude that there is a highly substantial relationship between the "Year of studying" and the Level of Self-Efficacy. The p-values for the parameters "Type of student accommodation" at 0.011 (χ2=6.525) and "Educational Background of the father" at 0.014 (χ2=4.707) are both less than 0.05, indicating statistical significance at a 5% level. Therefore, we can conclude that there is a notable connection between the "Type of student accommodation" and the "Educational Background of the father" and the "Level of Self-Efficacy" (Table 5).

**Table 5:** Association of self-efficacy with demographic variables ** - Significant at 1% level; * - Significant at 5% level; DF - Degree of freedom; BSc (N): BSc Nursing; DGNM: Diploma in General Nursing and Midwifery

S. No.	Demographic Variables	Class	Level of Self-Efficacy		Chi-square value	DF	P value
			Medium	High			
1	Age (in years)	15-20	39	29	1.413	1	0.235
		21-25	63	67			
2	Gender	Male	33	31	0.012	1	0.913
		Female	69	67			
3	Course	BSc Nursing	94	93	0.618	1	0.432
		DGNM	8	5			
4	Year of studying	BSc (N) 3rd year	49	42	96.255	4	0.000**
		BSc (N) 4th year	45	51			
		DGNM 2nd year	8	5			
5	Type of Student Accommodation	Hostel	35	18	6.525	1	0.011*
		Day Scholar	67	80			
6	Type of family	Nuclear family	68	71	1.353	1	0.245
		Joint family	32	23			
7	Order of child in the family	First child	57	57	0.220	2	0.896
		Second child	25	27			
		Third child	15	13			
8	Religion	Hindu	60	55	0.312	2	0.855
		Christian	31	33			
		Muslim	9	10			
9	Family Income	Less than 25,000	24	24	1.744	4	0.783
		25,000 to 50,000	32	25			
		50,000 to 75,000	17	14			
		75,000 to 1,00,000	21	24			
		Above 1,00,000	8	11			
10	Place of residence	Urban	62	61	0.064	2	0.969
		Semi-Urban	19	17			
		Rural	21	20			
11	Educational Background of the father	No formal education	10	4	16.022	6	0.014*
		Primary School Certificate	16	11			
		High School Certificate	26	39			
		Diploma	13	10			
		Graduate	22	23			
		Post Graduate	10	10			
		Others	5	1			
12	Educational Background of the mother	No formal education	9	7	4.707	5	0.453
		Primary School Certificate	23	22			
		High School Certificate	31	34			
		Diploma	10	3			
		Graduate	15	19			
		Post Graduate	12	10			
13	Occupation of father	Unemployed	5	1	4.378	4	0.357
		Semi-skilled Worker	11	11			
		Skilled Worker	29	33			
		Professional	31	35			
		Business	23	16			
		Others	3	2			
14	Occupation of mother	Unemployed/ Housewife	43	59	7.769	4	0.100
		Semi-skilled Worker	14	5			
		Skilled Worker	13	10			
		Professional	23	18			
		Business	6	6			

At the 1% level of significance, the p-values that correlate to "Age, Course, and Year of studying" are significantly lower than 0.01, at 0.001(χ2=13.661), 0.000 (χ2=149.595) and 0.006 (χ2=14.623) respectively. Consequently, it is possible to assert that there is a highly significant connection between the level of professional identity and the "Age", "Course", and "Year of study". Additionally, at the 5% level of significance, the p-value for "Type of student accommodation" is 0.043 (χ2=6.296), which is lower than the significance threshold of 0.05. Consequently, we may conclude that "Type of student accommodation" correlates considerably with the Level of Professional Identity (Table 6).

**Table 6:** Association between demographic variables and professional identity ** - Significant at 1% level; * - Significant at 5% level; DF - Degree of Freedom; BSc (N): BSc Nursing; DGNM: Diploma in General Nursing and Midwifery

S. No.	Demographic Variables	Class	Level of Professional Identity			Chi-square value	DF	P value
			Low	Moderate	High			
1	Age (in years)	15-20	26	15	28	13.661	2	0.001**
		21-25	38	55	38			
2	Gender	Male	26	22	18	2.959	2	0.228
		Female	38	49	49			
3	Course	BSc Nursing	61	65	63	149.595	2	0.000**
		DGNM	3	6	4			
4	Year of studying	BSc (N) 3rd year	34	20	39	14.623	4	0.006**
		BSc (N) 4th year	27	45	24			
		DGNM 2nd year	3	6	4			
5	Type of Student Accommodation	Hostel	17	25	11	6.296	2	0.043*
		Day Scholar	47	46	56			
6	Type of family	Nuclear family	39	49	52	4.647	2	0.098
		Joint family	24	18	14			
7	Order of child in the family	First child	39	41	35	2.582	4	0.630
		Second child	13	18	21			
		Third child	7	11	11			
8	Religion	Hindu	38	38	40	2.613	4	0.624
		Christian	18	27	19			
		Muslim	7	5	8			
9	Family Income	Less than 25,000	15	17	16	10.902	8	0.207
		25,000 to 50,000	25	15	18			
		50,000 to 75,000	7	9	15			
		75,000 to 1,00,000	12	22	12			
		Above 1,00,000	5	8	6			
10	Place of residence	Urban	36	46	42	3.756	4	0.440
		Semi-Urban	10	13	14			
		Rural	18	12	11			
11	Educational Background of the father	No formal education	5	5	4	2.173	10	0.995
		Primary School Certificate	9	9	9			
		High School Certificate	20	26	20			
		Diploma	9	7	7			
		Graduate	13	16	17			
		Post Graduate	6	6	8			
12	Educational Background of the mother	No formal education	4	2	10	17.886	10	0.057
		Primary School Certificate	16	19	10			
		High School Certificate	20	29	17			
		Diploma	7	2	4			
		Graduate	8	12	15			
		Post Graduate	7	6	9			
13	Occupation of father	Unemployed	2	3	1	7.895	8	0.444
		Semi-skilled Worker	6	5	11			
		Skilled Worker	22	23	18			
		Professional	18	28	20			
		Business	15	10	15			
14	Occupation of mother	Unemployed/ Housewife	26	42	36	12.975	8	0.113
		Semi-skilled Worker	10	6	3			
		Skilled Worker	10	7	6			
		Professional	15	11	15			
		Business	2	3	7			

## Discussion

The interplay between one's professional identity and self-efficacy is a vital factor in comprehending how individuals successfully navigate and prosper in their work surroundings. This study sought to elucidate the relationship between these two constructs, revealing significant insights into how one’s sense of professional identity may influence their perceived self-efficacy, and vice versa. By examining this correlation, our research aimed to contribute to the broader discourse on professional development and individual performance. In this section, we will interpret the implications of our findings and explore how they align with or challenge existing literature.

Demographic profile

In a study conducted by Xu et al. (2021), demographic profiles were polled for 656 vocational nursing students from two vocational colleges in Zhengzhou, China. This study revealed that the majority of the students, 624 (95.1%), were females, 367 (55.9%) were aged 21-25 years, 455 (69.3%) were from rural areas and 397 (60.51%) of their main guardian had the educational qualification of high school or secondary school [6].

These findings were very similar to the evaluation of demographic variables pertaining to nursing students conducted by us. It was revealed that out of the students surveyed, 131 (64.9%) were aged between 21-25 years, 136 (67.3%) students were females, 189 (93.6%) participants were in BSc Nursing (N) course, 96 (47.5%) subjects were from BSc (N) 4th year and 149 (73.8%) students were day scholars. Furthermore, 140 (69.3%) pupils belonged to nuclear families, 115 (56.9%) subjects were the first child, 116 (57.4%) subjects were Hindu, 58 (28.7%) participants had a family income of Rs 25,000 to 50,000, 124 (61.4%) pupils lived in urban areas, 66 (32.7%) of their fathers had achieved a high school certificate, 66 (32.7%) of their mothers had achieved a high school certificate, 66 (32.7%) of their fathers were professional and 104 (51.5%) of their mothers were housewife/unemployed.

Measuring the levels of professional identity and self-efficacy

Conducting an assessment of the professional identity and self-efficacy of student nurses enrolled at SRM College of Nursing constituted the first objective of the research. The assessment revealed that out of all the subjects scrutinized, 102 (50.5%) students demonstrated a medium degree of self-efficacy, 98 (48.5%) students showed a high level, and 2 (1.0%) students displayed a low level. Similarly, 71 (35.1%) students had a moderate level, 67 (33.2%) students had a high level, and 64 (31.7%) students had low levels of professional identity. An analogous outcome was observed in a research investigation carried out by Mei et al. (2021), wherein they graphed four self-efficacy profiles designated as Low, Medium-low, Medium-high, and High [4]. 

Relationship amidst professional identity and self-efficacy

Examining the relationship between professional identity as well as self-efficacy was the second objective of the study. A highly substantial positive association (r=0.489) was found between self-efficacy and professional identity according to Karl Pearson's coefficient of correlation. This positive correlation implied that nursing students with a prominent degree of self-efficacy have a compelling degree of professional identity and vice-versa.

A parallel outcome was observed in the study conducted by Xu et al. (2021), where academic self-efficacy (p < 0.05) and learning ability self-efficacy (p < 0.01) were both significantly connected with the professional attitude of higher vocational nursing students. This demonstrated that nursing students exhibited a more positive professional attitude when their academic self-efficacy was boosted. They stated that students who possess advanced cognitive abilities and a strong sense of self-efficacy have a trust in their own capacity to successfully accomplish learning activities. They demonstrate perseverance and diligence when faced with academic challenges and setbacks [6]. Furthermore, Wei et al. (2021) stated that the intention to stay in a profession and the job satisfaction levels are also affected positively when self-efficacy is developed properly [7].

Associating self-efficacy and professional identity with demographic variables

Associating levels of self-efficacy and professional identity with demographic variables was the third objective of the study. At the 1% level of significance, the p values that correlate to "Age, Course, and Year of studying" were significantly lower than 0.01, at 0.001 (χ2=13.661), 0.000 (χ2=149.595) and 0.006 (χ2=14.623) respectively. Consequently, it could be asserted that there exists a highly significant connection between the level of professional identity and the "Age", "Course", and "Year of study". It can be rationalized that Age, course, and year of study significantly shape professional identity by influencing experience, knowledge, and aspirations. Older students often bring prior work experience, fostering a clearer sense of purpose, while younger students may explore various career options. The specific course provides relevant skills and knowledge, while advancing through years of study builds confidence and specialization, helping students solidify their professional identity and align their goals with their chosen fields. Also, students in higher academic years are typically involved in networking, internships, and leadership roles, which solidify their professional identity and readiness for the job market.

Moreover, at the 5% level of significance, the p-value for "Type of student accommodation" was found to be 0.043 (χ2=6.296), which was lower than the significance threshold of 0.05. Consequently, we could conclude that "Type of student accommodation" correlated considerably with the Level of Professional Identity. Specifically speaking, students who were day scholars showed an increase in professional identity. A parallel finding was seen by Jafarianamiri et al. (2022), who reported that nursing students who expressed satisfaction with their work and resided with their families had a notably stronger professional identity [14]. It can be reasoned that day scholars can enhance their professional identity by actively engaging in campus activities, networking with peers and mentors, and gaining practical experience through internships. Building a personal brand online, setting career goals, and using commuting time for professional development are also possible measures that they can employ to build a strong professional identity.

The study of Ahlawat et al. (2022) reported that self-efficacy was linked with the ensuing socio-demographic factors: Academic Year level, Household Earnings, Residence Location, and career of the father [15]. This was comparable to our own study, where the p-value for the parameter "Year of studying" is 0.000 (χ2=96.255), which is below the threshold of 0.01, indicating a high degree of statistical significance at the 1% level. Therefore, we were able to conclude that there existed a highly substantial relationship between the "Year of Studying" and the Level of Self-Efficacy. It is plausible to argue that as students advance through the years, they encounter challenges that enhance their competence and resilience. This gradual mastery of subject matter and practical experiences leads to greater confidence in their abilities, reinforcing their belief in their capacity to succeed in academic and professional pursuits, ultimately boosting their self-efficacy.

We also found that the p-values for the parameters "Type of student accommodation" at 0.011 (χ2=6.525) and "Educational Background of the father" at 0.014 (χ2=4.707) are both less than 0.05, indicating statistical significance at a 5% level. Therefore, we can conclude that there is a notable connection between the "Type of student accommodation" and the "Educational Background of the father" and "Level of Self-Efficacy". It can be conjectured that day scholars can enhance their self-efficacy by setting clear, achievable goals and creating a structured routine that balances study and commute time. Actively engaging in campus activities, such as clubs and events, helps build skills and confidence. Building a supportive network of peers, mentors, and faculty provides encouragement and guidance, thereby enhancing their self-efficacy.

Implications for nursing education and practice

The study's findings permit us to draw the conclusion that nursing students require interventions to boost their sense of professional identity in addition to self-efficacy, and we may use these findings to broaden the scope of the nursing profession. Student nurses can develop self-efficacy as well as a professional identity through nursing education in order to enhance their academic performance, prepare themselves to confront the rigors of clinical environments, and increase their credibility and competence.

As members of the health team, nurses need to have a strong professional identity. A mass awareness program can be conducted on regular basis in many health centers to allow nurses to identify and ameliorate their self-efficacy and professional identity level.

To reduce the likelihood of employee turnover, nursing administration should work with influential groups to establish uniform rules and procedures that boost nurses' and students' sense of professional identity in addition to their self-efficacy. For the purpose of broadening medical professionals' skill sets, nursing administrators ought to assume leadership positions and inspire nursing staff to enhance their self-efficacy and professional identity.

Limitations

This research is cross-sectional in nature, thus precluding the establishment of causal relationships. Due to the fact that the study's sample is of Indian descent, its values and ideals may differ from those of other nations. Therefore, the implications cannot be extrapolated for diverse backgrounds. Professional identity may be influenced by supplementary characteristics; however they were not accounted for in the present investigation.

## Conclusions

In this investigation, researchers appraised the levels of self-efficacy in addition to professional identity levels amongst student nurses. A majority of the students demonstrated a moderate level of self-efficacy as well as professional identity. Further, a robust correlation was observed between the level of self-efficacy exhibited by student nurses and their professional identity. Additionally, substantial connections were derived between certain selected demographic parameters with self-efficacy and professional identity.

Future research can elaborate on the links between professional identity and self-efficacy and parameters such as age, course, year of study, and type of accommodation. Furthermore, student nurses can develop self-efficacy as well as a professional identity through nursing education in order to enhance their academic performance, prepare themselves to confront the rigors of clinical environments, and increase their credibility and competence. Subsequent studies can focus on providing interventions to student nurses in order to boost their sense of professional identity in addition to self-efficacy, and we may use these findings to broaden the scope of the nursing profession.
